# Putative Role of Tie2-Expressing Monocytes/Macrophages in Colorectal Cancer Progression Through Enhancement of Angiogenesis and Metastasis

**DOI:** 10.3390/cancers17172856

**Published:** 2025-08-30

**Authors:** Eman Amin M. Ali, Alaa Muayad Altaie, Iman M. Talaat, Rifat Hamoudi

**Affiliations:** 1 Research Institute of Medical and Health Sciences, University of Sharjah, Sharjah P.O. Box 27272, United Arab Emirates; u21105945@sharjah.ac.ae (E.A.M.A.); alaa.abed@sharjah.ac.ae (A.M.A.); 2 Center of Excellence for Precision Medicine, Research Institute of Medical and Health Sciences, University of Sharjah, Sharjah P.O. Box 27272, United Arab Emirates; 3 Clinical Sciences Department, College of Medicine, University of Sharjah, Sharjah P.O. Box 27272, United Arab Emirates; 4 Pathology Department, Faculty of Medicine, Alexandria University, Alexandria 21131, Egypt; 5 Division of Surgery and Interventional Science, University College London, London NW3 2PS, UK; 6 Biomedically Informed Artificial Intelligence Laboratory (BIMAI-Lab), University of Sharjah, Sharjah P.O. Box 27272, United Arab Emirates

**Keywords:** TEMs, colorectal cancer, angiogenesis, metastasis, progression

## Abstract

Colorectal cancer (CRC) is a major global health burden and a leading cause of cancer-related mortality, with metastasis representing the primary cause of death. Angiogenesis plays a critical role in this process, and macrophages within the tumor microenvironment (TME) are its key regulators. Among these, Tie2-expressing macrophages (TEMs) constitute a distinct pro-angiogenic subset that localizes to perivascular regions and responds to angiopoietin2 (Ang2) signaling. Moreover, TEMs contribute to vessel destabilization and the formation of permissive niches for cancer cell intravasation, linking them to both angiogenic and non-angiogenic modes of malignant tumor progression. The significance of TEMs in CRC remains controversial. This controversy, as we noticed, stems from a confluence of methodological challenges, lack of standardized markers, small-scale studies, inconsistent findings across studies, and the inherent complexity of both CRC biology and macrophage biology. Evidence from preclinical models and patient samples highlights the correlation between Ang2/Tie2 activity, TEM infiltration, and poor prognosis in CRC. This review summarizes current knowledge on the role of TEMs and the Ang/Tie2 axis in CRC angiogenesis, metastasis, and resistance to anti-angiogenic therapies. Advancing our understanding of TEMs may enable novel macrophage-focused strategies to inhibit CRC progression and improve patient outcomes.

## Simple Summary

Tie2-expressing monocytes/macrophages (TEMs) are a distinct subset of macrophages predominantly located near blood vessels. They play a critical role in promoting angiogenesis, as demonstrated in various experimental cancer models in mice. Furthermore, strong correlations between TEMs presence and disease stage have been observed in human solid tumors, such as gastric cancer, underscoring their significance in malignant tumor progression. This article highlights TEMs in colorectal cancer (CRC), a leading malignancy worldwide in both incidence and mortality, emphasizing their role as a distinct pro-angiogenic macrophage subset with critical functions in cancer progression and vascular biology, aiming to elucidate the role of TEMs in CRC tumor vascularization, metastasis, and potentially the response to anti-angiogenic therapies. Understanding the contribution of TEMs could pave the way for identifying novel macrophage-based prognostic biomarkers or therapeutic targets, with the potential to inhibit tumor progression and improve patient outcomes in CRC.

## 1. Introduction

### 1.1. Colorectal Cancer Background

Colorectal cancer (CRC) is one of the major health burdens worldwide, constituting 9.6% of registered cancer cases in 2022, according to GLOBOCAN data, following breast cancer and lung cancer. Additionally, CRC accounts for a significant number of deaths each year, estimated at 9.3% of total cancer deaths in 2022, with an expected doubling in incidence and deaths by 2040 [1].

CRC is a multifactorial disease influenced by a combination of genetic and environmental factors. Its pathogenesis is associated with several risk factors, which can be classified as non-modifiable factors such as advanced age, male sex, genetic predisposition, ethnicity, a positive family history, and hereditary mutations. On the other hand, modifiable risk factors are almost all related to unhealthy lifestyle options like smoking, excessive alcohol consumption, high and frequent red meat intake, inappropriate use of antibiotics that disturbs the normal balance of the colon microbiota, and the increased consumption of a westernized high-fat diet that is linked to increased body mass index and chronic low-grade inflammation [2].

Since CRC is highly lethal and associated with variable outcomes and drug responses due to heterogeneous gene expression profiles in different patients [3], it was necessary to resolve this issue. Therefore, in 2015, a comprehensive classification system of CRC known as the consensus molecular subtyping (CMS) emerged, providing great help in predicting prognosis and determining the most suitable treatments for CRC patients, though with some limitations in certain cases that do not fit within any of the categories included by the consensus classification system. CMS includes four groups (CMS1-CMS4), each with specific molecular characteristics, treatment options, and prognosis [4].

The transformation of normal colonic mucosa into malignant carcinoma that invades the basement membrane and metastasizes to distant organs typically follows the conventional adenoma-carcinoma sequence. This model progresses through two main molecular pathways: the chromosomal instability (CIN) pathway and the microsatellite instability (MSI) pathway. Alternatively, CRC can arise through the distinct serrated carcinoma pathway, also known as the CpG island methylator phenotype (CIMP), which is associated with a poorer prognosis compared to the other two pathways [5].

CRC deaths primarily result from metastasis [6], and since angiogenesis plays a crucial role in the metastatic process, investigating its key regulatory pathways can reveal novel strategies to enhance patient survival [7].

### 1.2. Tumor Microenvironment and Angiogenesis

Tumor angiogenesis is regulated by various components of the tumor microenvironment (TME), which includes all cellular and acellular constituents surrounding tumor cells. Over the last decade, it has become a focal point for research due to its significance in supporting tumor growth and its role as a link between cancer cells and the entire organism through vascular connections [8].

Angiogenesis is essential in solid tumors for nourishing the actively dividing malignant cells with nutrients and oxygen, as well as providing a pathway for cancer cells to spread to distant sites [9]. The activity of this vital process of new blood vessel formation from preexisting ones can be assessed by measuring the levels of several angiogenesis markers, which mainly include e-selectin, endostatin, fibroblast growth factor 2 (FGF2), matrix metallopeptidase-9 (MMP-9), and, most importantly, vascular endothelial growth factor-A (VEGF-A) [10].

In solid tumors, including CRC, tumor-associated macrophages (TAMs) are the primary cellular component in the TME. They generally exhibit anti-tumor activity through the M1 phenotype (classically activated), which can directly damage cancer cells by secreting reactive oxygen species and nitrogen species. Additionally, M1 TAMs can mediate antibody-dependent cellular cytotoxicity (ADCC) and activate T lymphocytes through cytokine production and antigen presentation. Conversely, pro-tumorigenic TAMs exhibit an M2 phenotype (alternatively activated), aiding tumor progression by supporting tumor cell proliferation, epithelial–mesenchymal transition, angiogenesis, invasion, metastasis, and immune suppression [11].

M1 and M2 states represent the extremes of a diverse array of macrophage polarization states, each characterized by a unique phenotype; some express highly proangiogenic factors such as vascular endothelial growth factor-A (VEGF-A) and matrix metalloproteinases (MMPs) [12].

Tumor angiogenesis primarily results from the angiogenic switch, the transition of endothelial cells (ECs) from a quiescent, inactive state to a proliferative condition. This process occurs when pro-angiogenic signals are stimulated by hypoxia, which upregulates the levels of hypoxia-inducible factor 1 (HIF-1), a transcription factor that enhances the expression of proangiogenic molecules by cancer cells and various cell populations in the TME. These include macrophages, neutrophils, myeloid-derived suppressor cells (MDSCs), eosinophils, Th2 (T helper 2), Th17 (T helper 17), mast cells, Tregs (T regulatory cells), B cells, NK (natural killer) cells, and platelets, as well as adipocytes and CAFs (cancer-associated fibroblasts). The increased levels of these molecules surpass angiostatic (anti-angiogenic) signals such as the basal lamina, low VEGFA gradients, low MMPs and cathepsin activity, Ang1 (angiopoietin1), THBS1 (thrombospondin 1), PAI1 (plasminogen activator inhibitor 1), and angiostatin [13]. Macrophages are the primary producers of numerous molecules that facilitate tumor angiogenesis, such as VEGFA, CXCL8 (chemokine (C-X-C motif) ligand 8), PlGF (placental growth factor), IL-1β (interleukin-1 beta), IL-6 (interleukin-6), TNF (tumor necrosis factor), MMPs, and cathepsins [14].

VEGFA binds to vascular endothelial growth factor receptor 2 (VEGFR2) on ECs, inducing division and migration towards prominent levels of VEGF. PlGF further exerts a mitogenic effect on ECs by binding to VEGFR2, while CXCL8 recruits neutrophils that secrete potent proangiogenic factors as well. Furthermore, IL-1beta, IL-6, and TNF activate downstream NF-kB signaling, increasing the expression of VEGFA and the extracellular matrix-degrading proteins matrix metalloproteases and cathepsins, which are crucial for endothelial cell migration and neoangiogenesis [9].

A special type of proangiogenic TAM is found to have a close, direct contact relationship with ECs. They are characteristically Tie2 (tyrosine kinase with immunoglobulin and epidermal growth factor (EGF) homology domains) receptor positive. Those Tie2+ TAMs, or Tie2-expressing macrophages (TEMs), secrete high levels of angiopoietin2 that mediate their interaction with ECs through EC Tie2 receptors [15]. Moreover, circulating TEMs show a highly proangiogenic expression profile when compared to Tie-negative ones; namely, they show higher levels of VEGFA, MMP-9, cyclo-oxygenase-2 (COX-2), and Wingless-related MMTV integration site family, member 5A (WNT5A), in addition to the elevated expression of cathepsin B and thymidine phosphorylase enzymes, which are known to be important in angiogenesis [16]. A summary of the roles of different angiogenesis regulators mentioned in Section 1.2 is available in Table 1.

TEMs were first identified in 2007 by Venneri and colleagues, who described a special type of macrophage that characteristically expresses the tyrosine kinase receptor (Tie2) and accounts for about 2–7% of circulating monocytes in the blood samples of healthy subjects. In cancer patients, it was detected in tumor tissues while sparing the adjacent normal areas, aligning with the in vitro findings that TEMs migrate towards Ang2 as a homing mechanism, with tumors expressing higher Ang2 in response to hypoxia. Their results also showed that TEMs constituted the main macrophage population alongside the well-known TAMs. They also found that mouse tumor xenograft models injected with human tumors exhibited markedly increased angiogenesis when TEMs were co-injected, but that effect was not observed with Tie2-monocytes [17].

**Table 1:** Summarizes the roles played by the different angiogenesis regulators in the tumor microenvironment (TME).

Factor	Role in Angiogenesis	Reference
E-selectin (Endothelial selectin)	Facilitates the adhesion of tumor cells to endothelial cells under shear stress. Soluble E-selectin activates FAK ^1^ signaling, increasing endothelial permeability and promoting extravasation.	[18]
Endostatin	Endogenous angiogenesis inhibitor; blocks VEGFR2 ^2^ signaling and integrin-mediated migration, leading to endothelial apoptosis and suppression of new vessel formation.	[19]
FGF2 (Fibroblast Growth Factor 2)	Binds FGFRs ^3^, activating MAPK/ERK ^4^ and PI3K/AKT ^5^ pathways in endothelial cells, stimulating proliferation, migration, and survival.	[20]
MMP-9 (Matrix Metalloproteinase-9)	Degrades extracellular matrix proteins, facilitates endothelial migration, and liberates matrix-bound VEGF-A ^6^ and growth factors, amplifying angiogenic signals.	[21]
VEGF-A (Vascular Endothelial Growth Factor-A)	Central angiogenic driver under hypoxia. Binds VEGFR2, inducing receptor dimerization and autophosphorylation, activating MAPK/ERK ^4^ and PI3K/AKT ^5^ signaling for endothelial proliferation and migration.	[22]
CXCL8/IL-8 (C-X-C motif chemokine ligand 8)/(Interleukin-8)	Binds CXCR1/2 ^7^ on endothelial cells, triggering PI3K/AKT ^5^ and MAPK/ERK ^4^ signaling. Recruits neutrophils, further enhancing angiogenesis.	[23]
PlGF (Placental Growth Factor)	Activates VEGFR1 ^8^, promotes endothelial cell proliferation and migration, and synergizes with VEGF-A ^6^ in neovascularization.	[24]
IL-1β (Interleukin-1β)	Activates NF-κB ^9^ in endothelial and tumor cells, inducing VEGF-A ^6^ and MMP-9 expression, promoting angiogenesis and vascular remodeling.	[9]
IL-6 (Interleukin-6)	Activates NF-κB ^9^ in endothelial and tumor cells, inducing VEGF-A ^6^ and MMP-9 expression, thereby promoting angiogenesis and vascular remodeling.	[9]
TNF-α (Tumor Necrosis Factor- α)	Activates NF-κB ^9^ in endothelial and tumor cells, inducing VEGF-A ^6^ and MMP-9 expression, thereby promoting angiogenesis and vascular remodeling.	[9]
Cathepsins (e.g., Cathepsin B)	ECM ^10^-degrading proteases enable endothelial invasion and release angiogenic growth factors from the extracellular matrix.	[25]
COX-2 (Cyclooxygenase-2)	Induces prostaglandin E2 (PGE2) production, which activates EP ^11^ receptors, stimulating VEGF-A ^6^ expression and angiogenic signaling in endothelial and tumor cells.	[26]
WNT5A (Wingless-related MMTV integration site family member 5A)	Activates non-canonical Wnt ^12^ signaling, enhancing endothelial cell migration, cytoskeletal rearrangement, and angiogenesis.	[27]
Thymidine phosphorylase	Catalyzes thymidine metabolism and generates angiogenic metabolites such as 2-deoxy-D-ribose, which promote endothelial cells migration and vessel formation.	[28]
Ang1 (Angiopoietin-1)	Binds Tie2 ^13^ receptor and stabilizes blood vessels by promoting endothelial cell proliferation, survival, and vessel barrier integrity.	[29]
Ang2 (Angiopoietin-2)	Context-dependent: blocks Ang1-Tie2 signaling to destabilize vessels and promote angiogenesis in the presence of VEGF-A ^6^; can also induce regression in the absence of VEGF-A ^6^.	[7]
THBS1 (Thrombospondin-1)	A potent anti-angiogenic protein; binds CD36 ^14^ and CD47 ^15^ on endothelial cells, triggering apoptosis and inhibiting VEGF-A signaling.	[30]
PAI-1 (Plasminogen Activator Inhibitor-1)	Inhibits plasminogen activators, blocking ECM degradation and angiogenesis.	[31]
Angiostatin	Endogenous angiogenesis inhibitor derived from plasminogen cleavage; interacts with surface molecules on ECs ^16^, inhibiting their migration and proliferation.	[32]

Abbreviations: ^1^ FAK: Focal Adhesion Kinase, ^2^ VEGFR2: Vascular Endothelial Growth Factor Receptor2, ^3^ FGFRs: Fibroblast Growth Factor Receptors, ^4^ MAPK/ERK: Mitogen-Activated Protein Kinase/Extracellular Signal-Regulated Kinase, ^5^ PI3K/AKT: Phosphoinositide 3-kinase/Protein Kinase B, ^6^ VEGF-A: Vascular Endothelial Growth Factor-A, ^7^ CXCR1/2: C-X-C Motif Chemokine Receptor 1 and C-X-C Motif Chemokine Receptor1/2, ^8^ VEGFR1: Vascular Endothelial Growth Factor1, ^9^ NF-κB: Nuclear Factor kappa B, ^10^ ECM: Extracellular Matrix, ^11^ EP receptors: Prostaglandin E Receptors, ^12^ Wnt: Wingless-Related Integration Site, ^13^ Tie2: Tyrosine Kinase With Immunoglobulin and EGF Homology Domains 2, ^14^ CD36: Cluster of Differentiation36, ^15^ CD47: Cluster of Differentiation47, ^16^ ECs: Endothelial Cells.

Nevertheless, the non-angiogenic progression of solid tumors, which refers to a growth pattern where tumors expand and invade surrounding tissues primarily by co-opting existing blood vessels rather than stimulating the formation of new vasculature through angiogenesis, is a well-recognized mechanism [33]. This process allows tumors to sustain themselves even in environments where angiogenic signals are weak or suppressed, making them less responsive to anti-angiogenic therapies [34]. Non-angiogenic growth has been observed in CRC, especially in CRC liver metastasis, where tumor cells migrate along pre-existing vessels, exploiting them for nutrients and oxygen. Those co-opting lesions have been observed to displace hepatocytes to access liver sinusoidal blood vessels by altering the hepatocytes phenotypically for further vessel co-option [35].

### 1.3. Tie2 Receptor Signaling

Angiopoietins, particularly the well-known Angiopoietin1 (Ang1) produced by pericytes and Angiopoietin2 (Ang2) produced by ECs, are ligands for the Tie2 receptor expressed almost exclusively on ECs and TEMs. This receptor plays an important role in regulating angiogenesis, with Ang-1 mediating vascular stability and maturation under normal physiological conditions. In contrast, Ang-2, especially at high VEGF levels produced in response to hypoxia, causes vessel destabilization and endothelial cell proliferation [36].

Tie2 receptors, when bound to Ang1, become autophosphorylated, which activates the PI3K/AKT pathway, resulting in endothelial cell survival, stabilization, and quiescence by enhancing the expression of survivin and eNOS while reducing the transcription of important apoptosis pathway components, namely caspase 9 and Bad. Moreover, Ang1/Tie2 binding leads to the inhibition of EC proliferation through the mitogen-activated protein kinase (MAPK) pathway, an effect mediated by both Grb2 and Dok-R, which bind to phosphorylated Tie2 at different sites. Ang1 binding to Tie2 on ECs can also suppress inflammation by inhibiting NF-kB through the IKK complex. While Ang2 at low concentrations acts as an antagonist to Ang1, competing with it and regulating its effect, at high concentrations, Ang2 can exert two opposite effects depending on the presence of VEGFA. In brief, when VEGFA is available, Ang2 promotes angiogenesis; however, it can cause blood vessel regression and shedding of perivascular cells when VEGFA is lacking, leading to vascular destabilization [37]. Tie2 receptors, as mentioned above, are also expressed by TEMs and interact with the same ligand, Ang2. Interestingly, despite TEMs using a homing mechanism to migrate from the circulation towards high Ang2 levels at tumor sites, these cells utilize Ang2/Integrin interactions for migration instead of the Ang/Tie2 axis [38].

Furthermore, TEMs are a crucial component of special tumor sites known as the tumor microenvironment of metastasis (TMEM), where Tie2 macrophages are in close direct contact with ECs, causing localized hyperpermeability that allows invasive, highly motile MENA+ (mammalian enabled protein) tumor cells to intravasate and metastasize [39]. Through live microscopy imaging in a mammary tumor mouse model, a transient and TMEM-exclusive vascular opening was observed. This phenomenon can be explained by the high VEGFA levels secreted by TEMs, which locally downregulate the endothelial junctions, causing their dissolution and increased leakiness [40].

In oncology, targeting the Ang/Tie2 pathway remains biologically compelling but clinically mixed: Ang-2 promotes vascular destabilization, hypoxia, and recruitment of pro-metastatic Tie2-high macrophages, while Tie2 activation can normalize tumor vessels, improving perfusion and drug delivery. Preclinical work with Ang2–binding, Tie2-activating antibodies (ABTAA) has demonstrated vessel normalization, reduced metastasis, and enhanced chemotherapy efficacy in multiple tumor models [41]. However, clinically, late-stage cancer trials of Ang-2 blockade alone—such as trebananib in recurrent ovarian cancer [42] and vanucizumab in metastatic colorectal cancer [43] have yielded limited benefit, with modest progression-free survival (PFS) gains or no improvement over anti-VEGF standards. These outcomes suggest that broad survival benefits are unlikely in advanced disease and that future promise lies in biomarker-guided use, early-stage settings, and rational combinations with anti-VEGF, immunotherapy, or macrophage-targeted agents.

Table 2, Table 3 and Table 4 list Ang/Tie2 targeting agents used in clinical trials, with brief mechanisms, stage of the study, and key findings in (1) ovarian cancer, (2) breast cancer, and (3) CRC, respectively, as the main three malignancies studied for the effect of Ang/Tie2 targeting compounds.

### 1.4. TEMs and Cancer

TEMs comprise a special, highly proangiogenic subpopulation of tumor-associated macrophages that reside in perivascular niches in tumors. Recruited by Ang2 under hypoxic conditions, these TEMs secrete elevated levels of pro-angiogenic mediators like VEGF-A and MMP-9. Their presence enhances tumor vascularization and supports angiogenic rebound following chemotherapy, contributing to relapses and disease progression. Notably, high densities of TEMs correlate with increased tumor micro-vessel density and poorer patient outcomes across several cancer types [49].

In an orthotopic mouse model of breast cancer and pancreatic neuroendocrine cancer, inhibition of the Tie2 receptor by the tyrosine kinase inhibitor Rebastinib remarkably reduced TEMs recruitment and showed a significant decrease in tumor angiogenesis, growth, and metastasis [46]. Moreover, Tie2 macrophages also showed a role in tumor relapse after chemotherapy in animal models, as deletion of the Tie2 receptor on macrophages conditionally caused a significant decrease in tumor revascularization and growth after chemotherapy [50].

In the context of non-angiogenic tumor growth, available studies showed a role of the Ang/Tie2 axis in vessel co-option but no evidence about the role of TEMs specifically. For example, Ang1 has been found to be involved in vascular co-option through unknown mechanisms [51], but recently, a positive correlation between Actin Related Protein 2/3 (Arp 2/3) complex expression and the presence of Ang1 in CRC liver metastasis has been revealed [52]. Ang1 serves as a positive regulator of ARP2/3 expression, which is a main enhancer of cancer cell motility. This positive regulation was noticed both in vitro and in vivo, playing a key role in the formation of vessel co-option in CRC liver metastases. Importantly, the expression of ARP2/3 driven by Ang1 was diminished in cancer cells following inhibition of Tie2 or the PI3K/AKT pathway in vitro. Overall, these findings reveal a new mechanism through which Ang1 facilitates vessel co-option in CRC liver metastasis, suggesting that targeting this pathway could provide promising therapeutic strategies to inhibit vessel co-option in these metastatic lesions [52].

On the other hand, it is established that co-opted vessels undergo regression mediated by Ang2, as it is known to induce vessel destabilization, particularly in the absence of VEGF-A. This vessel regression results in tumor cell loss and hypoxia, prompting the remaining tumor cells to adapt by switching to a neoangiogenic phenotype [53].

Overall, based on the discussed effect of Ang1 and Ang2, the two main ligands of the Ang/Tie2 axis on vessel co-option, together with the fact that TEMs are the main producers of Ang2 as discussed earlier, we can hypothesize that TEMs play a dual role in tumor progression. It might facilitate the hijacking of existing blood vessels through vessel destabilization mediated by Ang2 upregulation at low VEGF-A levels, enabling tumors to grow and invade surrounding tissues without triggering angiogenesis. Or, at high VEGF-A levels, it can support angiogenic tumor progression.

Another effect of TEMs detected in a murine mammary tumor model is that TEMs played a role in reducing the benefits of the vascular disrupting compound known as combrestatin A4 phosphate. The genetic deletion of TEMs or interruption of the CXCL12/CXCR4 axis, which is involved in TEM chemotaxis, resulted in significantly lower relapse rates and tumor neoangiogenesis after combrestatin treatment [54].

In humans, TEMs show variable significance in different types of cancer [55]. For example, in a study conducted on 76 patients newly diagnosed with gastric cancer, immunohistochemistry detection of Tie2 macrophages in tumor samples was directly correlated with TNM stage [56]. Surprisingly, in another study on 47 patients with hilar cholangiocarcinoma, Ang-1 levels and TEM infiltration were positively correlated with better prognosis and patient survival [57]. But this effect can be due to high Ang-1 levels that cause vascular stability and less tumor hypoxia, which is usually related to worse prognosis. Generally, TEMs are either derived from circulating monocytes or differentiated from TAMs at tumor sites. They have been found to be associated with tumor micro-vessel density, metastasis, and stage in a number of human solid tumors [55].

**Figure 1:**
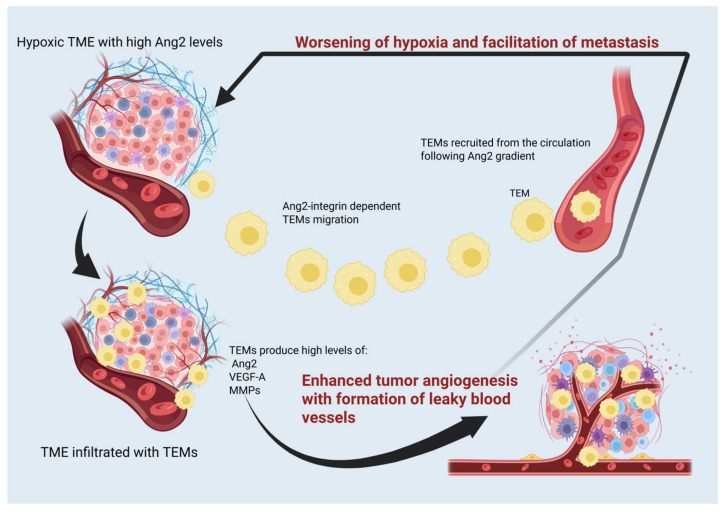
Tie2-expressing macrophage (TEM) infiltration of the tumor microenvironment (TME) and their effects on tumor angiogenesis and metastasis. High angiopoietin2 (Ang2) levels in hypoxic tumor tissue attract TEMs, which in turn perpetuate hypoxia and metastasis through the formation of incompetent blood vessels. VEGF-A: vascular endothelial growth factor-A, MMPs: matrix metalloproteases. Created using BioRender.com. https://app.biorender.com/illustrations/66570538e9fc3a8df4ccbfa0 (accessed on 28 July 2025).

The vicious cycle of tumor hypoxia, formation of incompetent, fragile blood vessels, leading to further worsening of hypoxia, is aggravated by TEM infiltration. They are a main source of Ang2 and VEGFA, as illustrated in Figure 1.

## 2. TEMs and CRC

It is important to note that, although the role of TEMs in cancer-related angiogenesis and metastasis has been extensively studied across various malignancies [55], research specifically addressing their function in CRC remains limited. Most existing studies tend to focus on the angiopoietin/Tie2 signaling axis in CRC more broadly, without explicitly delineating the specific contribution of TEMs.

### 2.1. Significance of Ang/Tie2 Axis in CRC

In CRC patients, immunohistochemistry (IHC) detection of Ang2, Tie2, and downstream molecules PI3K and AKT showed significant correlation with tumor stage and differentiation level, and their levels were significantly higher in tumor tissues compared to healthy adjacent tissues [58]. Moreover, high Ang2 levels measured by IHC correlated with poor overall survival in CRC patients, indicating the high importance of the Ang/Tie2 axis in CRC progression [59]. While another IHC-based study showed that Ang2, Tie2, and VEGFR2 expression show positive correlation with the CRC tumor marker carcinoembryonic antigen (CEA) and microvascular density (MVD), Ang2, but not Tie2 or VEGFR2 levels, correlates with the carbohydrate antigen19-9 (CA 19-9) CRC tumor marker [60]. Furthermore, plasma levels of Ang2 and Tie2, but not Ang1, showed significantly higher values as measured by enzyme-linked immunosorbent assay (ELISA) in a cohort of CRC patients compared to healthy controls, with Ang2 specifically showing markedly higher plasma levels in stage 3 compared to stage 2 CRC disease patients [61].

High Ang2 levels contribute to tumor progression and worse outcomes in CRC by destabilizing existing blood vessels, disrupting vascular maturation, and enabling enhanced angiogenesis, especially in cooperation with VEGF. Elevated Ang-2 has also been shown to correlate with more aggressive tumor behavior, such as increased proliferation, invasion, and metastasis, and serves as an independent prognostic marker for poorer survival [62,63].

In LoVo CRC cells, Ang2 knockdown impaired proliferation, migration, and invasion, while overexpression was associated with advanced tumor stages, vascular/lymphatic invasion, and metastasis, highlighting its role in aggressiveness and progression [62].

In animal models, inhibition of Ang/Tie2 interaction in the COLO205 (human CRC cell line) xenograft mouse model using trebananib (AMG 386) caused inhibition of the proliferation of tumor-associated ECs [64], matching results of the inhibition of Ang2/Tie2 receptor interaction by a specific peptide known as CovX-Bodies in xenograft mice models injected subcutaneously also with the colon cancer cell line COLO205, which caused significantly reduced tumor vascular density and tumor growth, together with reduced frequency of tumor-infiltrating Tie2+ macrophages compared to controls [65]. In addition, Western blotting showed markedly higher expression levels of Ang1, Ang2, Tie2, and VEGF in the colorectum of the CRC rat model compared to the normal rats in the control group [66].

Clinically, patients with high serum Ang2 had significantly worse overall survival in stage IV CRC, making it a robust prognostic biomarker [67]. And mechanistically, in metastatic CRC, Ang2 opposes vascular normalization by anti-VEGF therapy, undermining therapeutic benefit and contributing to poorer outcomes [63].

Ang1 and angiopoietin2 also play important but contrasting roles in vessel co-option. Ang1 primarily promotes vessel stability and maturation by binding to the Tie2 receptor on endothelial cells, which supports the maintenance of existing blood vessels and prevents their regression. In the context of vessel co-option, Ang1 helps maintain the integrity of the pre-existing vasculature that tumor cells exploit for growth. Conversely, Ang2 acts as a vessel-destabilizing cytokine, also binding to Tie2 but generally promoting vascular remodeling, sprouting, or regression depending on the presence of other factors like VEGF. In vessel co-option, Ang2 facilitates the detachment and regression of co-opted vessels, leading to hypoxia and tumor adaptation [52,53]. Together, these angiopoietins regulate the delicate balance between vessel stability and destabilization, which is crucial for the process of vessel co-option in tumor progression.

**Figure 2:**
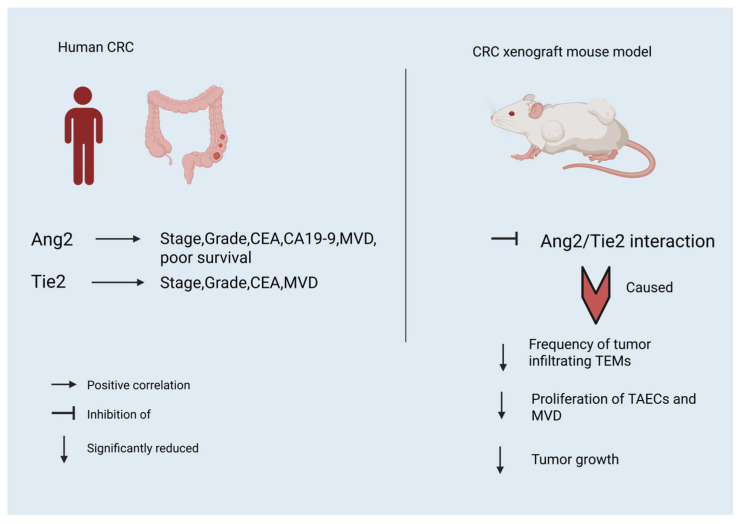
Significance of Ang2 and Tie2 (tyrosine kinase with immunoglobulin and epidermal growth factor (EGF) homology domains) receptor levels in human CRC patients, and significance of Ang2/Tie2 interactions and TEMs infiltration in CRC xenograft mouse models. (CA19-9: carbohydrate antigen19-9 tumor marker, CEA: carcinoembryonic antigen tumor marker, MVD: micro-vessel density, TAECs: tumor-associated endothelial cells). Created with BioRender.com. https://app.biorender.com/illustrations/66582cc0a7f9c88339b2ef0b (accessed on 28 July 2025).

Collectively, the available studies indicate that the Ang/Tie2 axis and TEMs play a major role in CRC progression both in humans and in CRC xenograft mouse models (Figure 2).

Considering the undeniable importance of the Tie2 receptor in CRC, we carried out an extensive review of the literature and data from the Catalogue of Somatic Mutations in Cancer (COSMIC) to investigate the presence of any Tie2 mutations linked to CRC, and our search revealed no evidence of Tie2 receptor mutations associated with colorectal cancer CRC pathogenesis. Instead, Tie2 mutations have predominantly been linked to venous malformations [68,69], cutaneomucosal venous malformations [70], and glaucoma [71].

**Figure 3:**
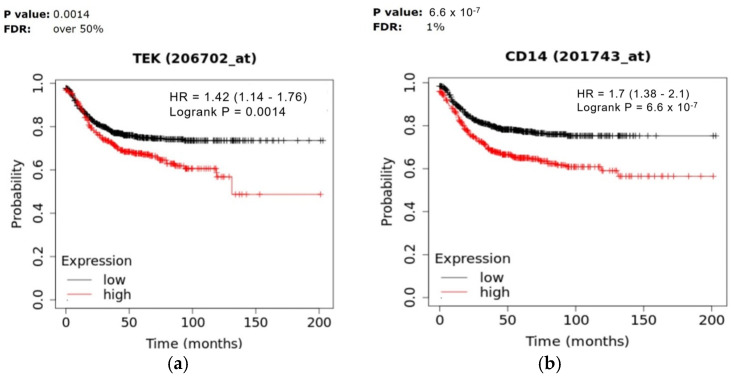
Kaplan–Meier plotter results for the (**a**) Tie2(TEK) gene, and (**b**) for the CD14 gene. HR: Hazard Ratio, FDR: False Discovery Rate.

To further explore the clinical relevance of TEMs in CRC, the correlation between the expression of TEM-associated markers, specifically TEK (the gene encoding Tie2) and CD14, with patient survival was analyzed using the Kaplan–Meier Plotter platform [72]. The analysis revealed that higher TEK expression was significantly associated with reduced survival in CRC patients (*p* = 0.0014; Figure 3a). Similarly, elevated CD14 expression correlated with significantly poorer survival outcomes (*p* = 6.6 × 10^−7^; Figure 3b). These findings underscore the potential prognostic value of the Tie2 receptor and, by extension, TEMs in CRC.

### 2.2. Infiltrating Immune Cell Composition of Tie2+/CD14+ CRC

**Figure 4:**
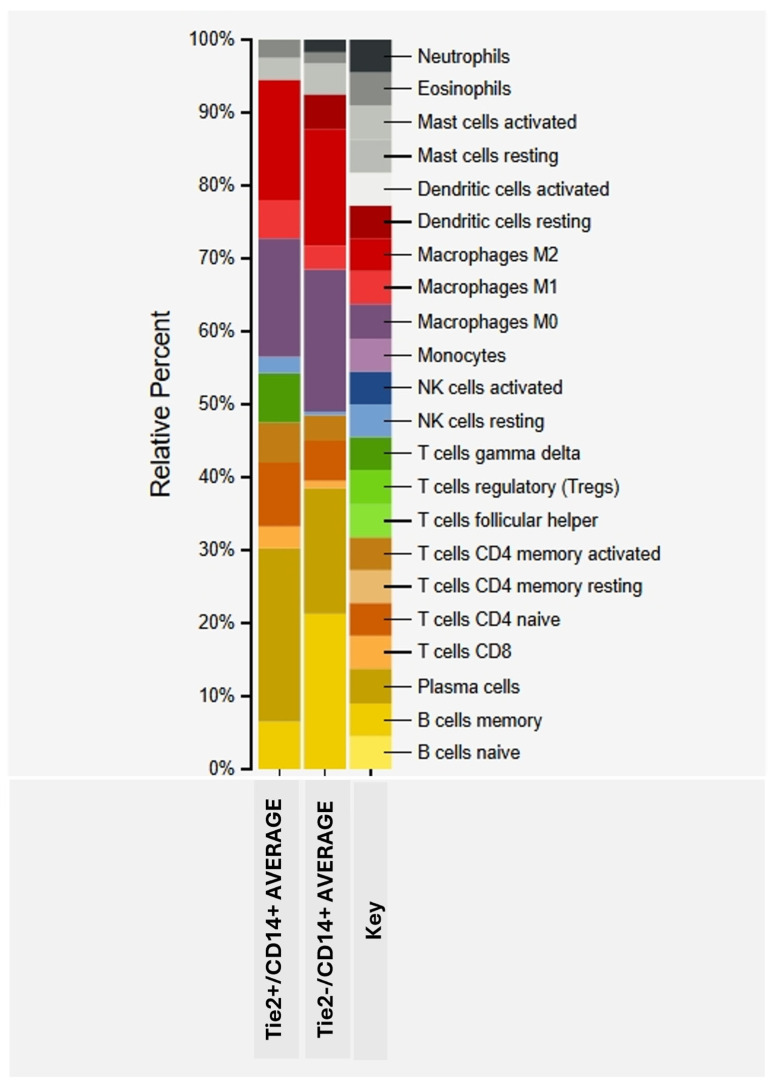
Percentages of different immune cell types, each represented by a color, as in the key included. Obtained from the CIBERSORTx tool analysis results of Tie2+/CD14+ and Tie2−/CD14+ groups’ average gene expression data.

To explore the differences in immune cell infiltration between Tie2^+^/CD14^+^ and Tie2^−^/CD14^+^ CRC tissues, the CIBERSORTx analytical tool [73] was employed to assess 22 transcriptomic profiles (10 Tie2^+^/CD14^+^ and 12 Tie2^−^/CD14^+^) retrieved from the publicly available Gene Expression Omnibus (GEO) dataset GSE41568 [74]. Sample selection was based on median-centered expression values, using the median as a cutoff point for both TEK (Tie2) and CD14 gene expression levels independently. Results showed that in the Tie2+/CD14+ group, 6.8% of the immune cells in CRC tissues are γδ T lymphocytes. While in the Tie2−/CD14+, no γδ T cells were detected (Figure 4, Table S1).

γδ T cells are a subset of circulating lymphocytes and constitute a small proportion of the TME population. They are distinguished by their heterodimeric γδ T cell receptor, and most of them are CD4- or CD8- cells, in contrast to the more abundant regular alpha beta αβ T cells, which express CD4 or CD8 surface markers. γδ T cells under physiological conditions form part of the gut intraepithelial lymphocytes and are known for their surveillance function, as they act as a first line of defense against pathogens and malignant transformations [75]. Gamma delta (γδ) T cells can recognize and attack CRC cells with potent killing mechanisms, like direct cytotoxicity or activation of death receptor signaling, as examples. Thereby suppressing tumor progression [75,76].

Nowadays, a major concern is growing about γδ T cells, especially the γδ T17 subset in CRC, as their counts positively correlate with the TNM stage, tumor size, tumor invasion, lymphatic and vascular invasion, lymph node metastasis, and the serum CEA level [77,78]. Those correlations can be directly explained by the fact that γδ T17 cells secrete considerable amounts of IL-17 cytokine, which can perpetuate CRC progression via promoting angiogenesis and expansion of MDSCs [79].

Whether γδ T cells in the Tie2+/CD14+ group are of the γδ T17 phenotype or not, in addition to their possible interaction with TEMs, needs further investigation.

On the other hand, the Tie2−/CD14+ group shows a considerably higher percentage of memory B cells (21%), compared to 6.4% in the Tie2+/CD14+. The role of memory B cells in CRC is complex and can vary according to the overall immune landscape of the patient [80]. Generally, the presence of memory B cells in the TME of CRC suggests an ongoing immune response against the tumor, as they can differentiate into plasma cells, which produce antibodies that can target and potentially kill tumor cells, and some studies suggest that the memory B phenotype might be associated with better outcomes in CRC [81]. Of note, it is proven that memory B cells are involved in tumor progression of different types of cancer, including CRC, through various mechanisms. For example, it is able to differentiate into regulatory B cells, which can suppress the activity of other immune cells, including T cells, that are important for fighting malignant tumors [82].

Interestingly, there is no noticeable difference in the percentages of M0, M1, and M2 macrophages between Tie2+/CD14+ and Tie2−/CD14+ groups as seen in Figure 3 and Table S1. This finding can be explained by the highly related gene expression profiles of TEMs and TAMs, despite the significantly higher proangiogenic and tissue remodeling signature and lower proinflammatory gene expression of TEMs [83].

Regarding the important antigen-presenting cells, dendritic cells (DCs), both groups (Tie2+/CD14+ and Tie2−/CD14+) lack activated (mature) DCs, which are known to enhance antitumor immunity [84]. The Tie2−/CD14+ group has 4.8% resting (immature) DCs that are characterized by their relative inability to present the antigens they engulf to T cells and are considered tolerogenic [84]. Ironically, immature DCs in tumor tissues have been found to enter an intrinsic activation process, followed by upregulation of chemokine receptor 7 (CCR7), for improved motility towards lymph nodes, where they can present tumor antigens efficiently [85]. Intriguingly, a study by Kocián, P. et al. showed that CRC patients with higher immature DCs and lower activated DC infiltration have higher tumor relapse rates [86]. This functional plasticity of DCs represents a challenge to concluding the role of each type of DC in the TME [87,88].

### 2.3. Presence of TEMs in CRC Tumor Tissues

In the 2007 study that first identified TEMs in human cancers, flow cytometric analysis of tumor-infiltrating monocytes in CRC samples revealed that approximately 55% of CD14^+^ monocytes expressed Tie2. Moreover, the expression level of Tie2 receptors in tumor-infiltrating TEMs was significantly higher than that observed in circulating TEMs from peripheral blood. Notably, Tie2^+^/CD45^+^ hematopoietic cells were largely absent in adjacent normal tissues. These findings were confirmed by immunohistochemistry and confocal immunofluorescence staining, further validating the presence and tumor-specific enrichment of TEMs in CRC [17].

A study involving 40 treatment-naïve CRC patients and 17 healthy controls reported the presence of TEMs in both tumor tissue and peripheral blood of CRC patients. However, TEM counts showed no significant association with tumor vascularity, disease stage, or response to anti-angiogenic therapy. Notably, Tie2 expression levels on monocytes were significantly elevated in CRC patients compared to healthy controls [89], suggesting that Tie2 expression on circulating monocytes may play a role in the early stages of CRC development. Nevertheless, the limited sample size (fewer than 100 subjects), combined with the absence of molecular characterization such as MSI or mutational status, which are known to influence immune cell infiltration in CRC [90], limits the generalizability of these findings across broader CRC patient populations.

Interestingly, a single-cell transcriptomic analysis involving samples from 210 patients across 15 different cancer types, including CRC, aimed to identify distinct subsets of tumor-infiltrating myeloid cells. This study identified secreted phosphoprotein 1 (SPP1^+^) macrophages as the only proangiogenic TAMs detected in CRC [91]. However, these findings may be limited by the broad scope of the analysis, which included multiple tumor types rather than focusing specifically on CRC. It is plausible that additional subsets of proangiogenic monocytes/macrophages, including Tie2-expressing monocytes/macrophages (TEMs), may emerge with more focused single-cell studies that exclusively examine CRC samples [92].

In a separate investigation, another research group questioned the significance of TEMs within the broader population of tumor-infiltrating myeloid cells (TIMs). To address this, they conducted a meta-analysis using integrated single-cell transcriptomic data from publicly available human and mouse datasets across 13 cancer types. Their analysis revealed that the frequency of Tie2^+^ TIMs was generally low, with a maximum of 0.219% observed in human lung adenocarcinoma and 1.099% in a mouse melanoma model. In the case of CRC, Tie2^+^ TIMs were found to be below the detection threshold [92].

It is important to note that the single-cell RNA sequencing analyses in the aforementioned studies were conducted across multiple cancer types using a limited number of datasets. These analyses primarily focused on myeloid cells, without specifically isolating or characterizing the monocyte/macrophage populations. This lack of focus may account for the minimal representation of Tie2^+^ macrophages observed in their findings, both generally and in CRC specifically. Consequently, further investigation is warranted using larger, CRC-focused single-cell datasets to accurately assess the presence and frequency of Tie2^+^ monocytes/macrophages within the colorectal tumor microenvironment.

### 2.4. TEMs and CRC Metastasis

A recent murine study investigated the role of Src homology-2 containing protein tyrosine phosphatase (SHP2), a negative regulator of Tie2 receptor signaling, in modulating the function of TEMs via the angiopoietin (Ang)/Tie2-phosphatidylinositol-3-kinase (PI3K)/protein kinase B (Akt)/mammalian target of rapamycin (mTOR) signaling cascade. Using SHP2-deficient mice, an in vivo model of CRC liver metastasis was established. The results demonstrated that SHP2-deficient mice developed significantly more metastatic nodules on the liver surface compared to wild-type controls. These findings suggest that increased activation of the Ang/Tie2–PI3K/Akt/mTOR pathway in TEMs may enhance tumor-associated microangiogenesis and promote CRC liver metastasis [93].

The Tie2 receptor is classified among the key immunokinases—alongside colony-stimulating factor 1 receptor (CSF1R) and VEGFR2—that contribute to the establishment of an immunosuppressive TME in CRC. Inhibition of these kinases using SJ-C1044 in CRC xenograft models harboring KRAS and BRAF mutations has been shown to reprogram the TME toward a more immunopermissive and antitumorigenic state. This was evidenced by enhanced T cell infiltration and a concurrent reduction in TAMs and regulatory T cells (Tregs) [94]. These observations suggest that the Tie2 receptor may facilitate metastatic progression by modulating the immune landscape of the tumor microenvironment (TME). An immunosuppressive TME is well-established as a critical enabler of all stages of the metastatic process [95,96].

Additionally, the Tie2 receptor has been identified as a marker of alternatively activated (M2-like) macrophages, which are detectable in CRC [95]. This association further supports the hypothesis that TEMs may promote CRC progression not only through their characteristic perivascular proangiogenic activity but also via mechanisms attributed to M2 macrophages, including immunosuppression, tissue remodeling, and support of tumor growth.

### 2.5. TEMs and Anti-Angiogenic Therapy Response in CRC

Based on the high impact of the Ang/Tie2 axis and TEM populations on anti-angiogenic therapy response, we hypothesize that monitoring changes in TEM blood counts along with Ang2 levels before and during anti-angiogenic therapy across large cohorts of CRC patients at various disease stages might yield a valuable predictive biomarker for treatment response and overall benefit. Despite the established function of TEMs in promoting tumor angiogenesis, only a few small-scale clinical studies have directly assessed their blood count changes in response to targeted anti-angiogenic interventions.

In a clinical study involving 60 patients with metastatic CRC, the effects of bevacizumab—a monoclonal antibody targeting VEGFon circulating monocyte subsets were examined. The findings indicated no significant changes in the levels of intermediate monocytes or TEMs following treatment [96]. However, the absence of intra-tumoral analysis limits the conclusions that can be drawn, as changes in TEM infiltration at tumor sites may not be reflected in peripheral blood.

Notably, elevated intra-tumoral Ang2 is recognized as a key resistance mechanism to anti-angiogenic therapy, acting through the Tie2 receptor to sustain angiogenesis despite VEGF blockade [97]. Therefore, assessing Ang2 expression levels in the tumor microenvironment may provide more clinically relevant insights into therapeutic response than monitoring peripheral monocyte counts alone.

Increasing evidence suggests that the mutational landscape of CRCs significantly influences response to anti-angiogenic therapy. For example, tumors harboring KRAS mutations often exhibit intrinsic resistance to single-agent anti-angiogenic therapy, a resistance thought to be mediated in part by Ang2 upregulation. This supports the rationale for employing combination anti-angiogenic strategies in KRAS-mutant CRC to overcome such resistance and improve therapeutic efficacy [98].

Additionally, the impact of vessel co-option in anti-angiogenic therapy resistance cannot be overlooked. Recognizing this alternative mode of tumor progression is crucial for developing more effective treatment strategies, as it highlights mechanisms of resistance and tumor adaptability beyond traditional angiogenesis dependency.

The hypothesized combined effect of TEMs in both angiogenic and nonangiogenic progression might have a role in enabling tumors to evade anti-angiogenic therapies and contribute to tumor resilience and metastasis.

## 3. Conclusions

TEMs represent a subset of myeloid cells with highly proangiogenic activity at tumor sites. Their presence correlated with tumor vascularity, growth, and metastasis in different human cancers, with up-to-date data obtained from studies on CRC xenograft mouse models providing strong evidence of similar effects in CRC patients supported by the positive correlation of TEM-related signaling molecules such as Tie2 and its downstream molecules, namely PI3K and AKT levels, and CRC disease stage, grade, MVD, and overall survival in human CRC patients. This highlights the possibility of involvement of TEMs in CRC progression through the formation of incompetent blood vessels that worsen tumor hypoxia and, at the same time, allow easier tumor cell intravasation and metastasis. Whether TEMs contribute to CRC progression through other mechanisms, such as modulating the immune status of the tumor microenvironment, or if they have direct communication with tumor cells that promote proliferation or metastasis, remains to be elucidated.

## 4. Limitations and Future Perspectives

Current understanding of the role of Tie2-expressing monocytes/macrophages (TEMs) in colorectal cancer (CRC) remains limited by several factors. Most evidence derives from preclinical models or small patient cohorts, and the phenotypic definition of TEMs is not fully standardized, making cross-study comparisons difficult. Functional heterogeneity within the TEM population, as well as their dynamic plasticity under different microenvironmental cues, complicates the interpretation of their precise contribution to CRC angiogenesis and metastasis. Furthermore, it remains unclear whether TEM infiltration is a driver of disease progression or a byproduct of worsening tumor hypoxia and rising Ang2 levels. Clinically, there is a lack of large-scale prospective studies linking TEM density or Tie2 signaling activity to patient outcomes, and no validated biomarkers currently guide TEM-targeted interventions. From a therapeutic standpoint, selectively modulating TEM activity without impairing beneficial macrophage immune responses poses a major challenge.

Future research should focus on multi-omics profiling of TEMs in CRC to define context-specific signatures. The development of in vivo imaging or circulating biomarkers for CRC patient stratification and the integration of TEM-targeted strategies—such as Tie2 kinase inhibitors, macrophage reprogramming agents, or vessel-normalizing therapies—into combination regimens with chemotherapy, anti-VEGF agents, or immunotherapy can be more informative about the results of TEM-targeted approaches, to determine whether targeting TEMs can meaningfully suppress angiogenesis, limit metastasis, and improve outcomes in CRC.

## Figures and Tables

**Table 2:** Summarizes clinical trials that targeted Ang/Tie2 pathway in ovarian cancer.

Candidate Drug	Modality/Target	Mechanism	Model/Setting	Stage/Status	Key Findings	Reference
Trebananib (AMG 386)	Peptibody neutralizing Ang ^1^ & Ang2 ^2^	Ligand trap; prevents Ang1/2 binding to Tie2 ^3^ → anti-angiogenic	Phase III TRINOVA-1 ^4^: recurrent OC ^5^ + weekly paclitaxel	Phase III completed	Improved PFS ^6^; no OS ^7^ benefit Acceptable safety	[44]
MEDI3617	mAb ^8^ vs. Ang2	Ang2 neutralization (relieves Tie2 inhibition)	Phase I in advanced tumors with ovarian cancer cohorts	Phase I completed	Overall lack of clinical efficacy was observed Generally tolerable	[45]

Abbreviations: ^1^ Ang1: Angiopoietin1, ^2^ Ang2: Angiopoietin2, ^3^ Tie2: Tyrosine Kinase with Immunoglobulin and EGF (Epidermal Growth Factor) Homology Domains, ^4^ TRINOVA-1: Anti-Angiopoietin Therapy with Trebananib for Recurrent Ovarian Cancer Trial, ^5^ OC: Ovarian Cancer, ^6^ PFS: Progression-Free Survival, ^7^ OS: Overall Survival, ^8^ mAb/Monoclonal Antibody.

**Table 3:** Summarizes clinical trials that targeted Ang/Tie2 pathway in breast cancer.

Candidate Drug	Modality/Target	Mechanism	Model/Setting	Stage/ Status	Key Findings	Reference
Rebastinib (DCC-2036) + paclitaxel/eribulin	Small-molecule Tie2 ^1^ kinase inhibitor	Blocks Tie2 high macrophages, reduces TMEM ^2^ function	Phase Ib in HER2 ^3^-negative MBC ^4^	Phase Ib/II ongoing	TMEM inhibition; Circulating tumor cells decreased significantly with the combined treatment, generally tolerable	[46,47]

Abbreviations: ^1^ Tie2: Tyrosine Kinase with Immunoglobulin and EGF (Epidermal Growth Factor) homology domains, ^2^ TMEM: Tumor Micro-Environment of Metastasis, ^3^ HER2: Human Epidermal Growth Factor Receptor 2, ^4^ MBC: Metastatic Breast Cancer.

**Table 4:** Summarizes clinical trials that targeted Ang/Tie2 pathway in colorectal cancer (CRC).

Candidate Drug	Modality/Target	Mechanism	Model/Setting	Stage/Status	Key Findings	Reference
Vanucizumab (RG7221) + mFOLFOX-6 ^1^	Bispecific mAb ^2^ against VEGF-A ^3^ & Ang2 ^4^	Dual ligand blockade	Phase II McCAVE ^5^ trial in mCRC ^6^	Phase II completed	No PFS ^7^ improvement vs. bevacizumab	[43]
Nesvacumab (REGN910)	mAb ^2^ vs. Ang2 ^4^	Selective Ang2 ^4^ blockade	Phase I in advanced solid tumors	Phase I completed	Preliminary antitumor activity was observed in patients with treatment-refractory advanced solid tumors	[48]

Abbreviations: ^1^ mFOLFOX-6: modified FOLFOX-6, FOLFOX-6: a chemotherapy regimen that includes the drugs (5-Fluorouracil, Leucovorin (Folinic acid), and Oxaliplatin, ^2^ mAb: monoclonal antibody. ^3^ VEGF-A: vascular endothelial growth factor-A, ^4^ Ang2: angiopoietin2, ^5^ McCAVE: vanucizumab plus mFOLFOX-6 versus bevacizumab plus mFOLFOX-6, ^6^ mCRC: metastatic CRC, ^7^ PFS: progression-free survival.

## Supplementary Materials

The following supporting information can be downloaded at https://www.mdpi.com/article/10.3390/cancers17172856/s1: Table S1: CIBERSORTx output display.
