# Supplementary material for: Putative Role of Tie2-Expressing Monocytes/Macrophages in Colorectal Cancer Progression Through Enhancement of Angiogenesis and Metastasis
Source: Cancers (Basel). 2025 Aug 30;17(17):2856. doi: 10.3390/cancers17172856 (PMC12427574; doi:10.3390/cancers17172856)
Supplement: Supplementary file 1 [file cancers-17-02856-s001.zip › cancers-3814394-supplementary.pdf]

Table S1. CIBERSORTx output display, showing the percentages of different immune cells populations in Tie2+/CD14+ and Tie2-/CD14+ groups and the p < 0.0001 in both groups.

Input Sample	B cells naive	B cells memory	Plasma cells	T cells CD8	T cells CD4 naive	T cells CD4 memory resting	T cells CD4 memory activated	T cells follicular helper	T cells regulatory (Tregs)	T cells gamma delta	NK cells resting	NK cells activated	Monocytes	Macrophages M0	Macrophages M1	Macrophages M2	Dendritic cells resting	Dendritic cells activated	Mast cells resting	Mast cells activated	Eosinophils	Neutrophils	p-value
Tie2+/CD14+ AVERAGE	0	0.064	0.239	0.03	0.086	0	0.056	0	0	0.068	0.021	0	0	0.164	0.053	0.164	0	0	0	0.03	0.024	0.000	0.000
Tie2-/CD14+ AVERAGE	0	0.212	0.172	0.01	0.056	0	0.036	0	0	0	0.005	0	0	0.194	0.033	0.159	0.048	0	0	0.043	0.013	0.018	0.000
